# High-efficient production of mushroom polyketide compounds in a platform host *Aspergillus oryzae*

**DOI:** 10.1186/s12934-023-02071-9

**Published:** 2023-03-30

**Authors:** Haiyan Han, Chunyan Yu, Jianzhao Qi, Pengchao Wang, Peipei Zhao, Wenbing Gong, Chunliang Xie, Xuekui Xia, Chengwei Liu

**Affiliations:** 1 Key Laboratory for Enzyme and Enzyme-Like Material Engineering of Heilongjiang, College of Life Science, Northeast Forestry University, Harbin, 150040 Heilongjiang China; 2 Biology Institute, Qilu University of Technology (Shandong Academy of Sciences), Jinan, 250103 Shandong China; 3 Institute of Bast Fiber Crops, Chinese Academy of Agricultural Sciences, Changsha, 410205 Hunan China

**Keywords:** Orsellinic acid, *o*-Orsellinaldehyde, Polyketide synthase, Basidiomycetes, *Aspergillus oryzae*

## Abstract

**Background:**

Orsellinic acid (2,4-dihydroxy-6-methylbenzoic acid, OA) and its structural analog *o*-Orsellinaldehyde, have become widely used intermediates in clinical drugs synthesis. Although the research on the biosynthesis of such compounds has made significant progress, due to the lack of suitable hosts, there is still far from the industrial production of such compounds based on synthetic biology.

**Results:**

With the help of genome mining, we found a polyketide synthase (PKS, HerA) in the genome of the *Hericium erinaceus*, which shares 60% amino acid sequence homology with ArmB from *Armillaria mellea*, an identified PKS capable of synthesizing OA. To characterize the function of HerA, we cloned *herA* and heterologously expressed it in *Aspergillus oryzae*, and successfully detected the production of OA. Subsequently, the introduction of an incomplete PKS (Pks5) from *Ustilago maydis* containing only three domains (AMP-ACP-R), which was into *herA*-containing *A. oryzae*, the resulted in the production of *o*-Orsellinaldehyde. Considering the economic value of OA and *o*-Orsellinaldehyde, we then optimized the yield of these compounds in *A. oryzae*. The screening showed that when maltose was used as carbon source, the yields of OA and *o*-Orsellinaldehyde were 57.68 mg/L and 15.71 mg/L respectively, while the yields were 340.41 mg/Kg and 84.79 mg/Kg respectively in rice medium for 10 days.

**Conclusions:**

Herein, we successfully expressed the genes of basidiomycetes using *A. oryzae* heterologous host. As a fungus of ascomycetes, which not only correctly splices genes of basidiomycetes containing multiple introns, but also efficiently produces their metabolites. This study highlights that *A. oryzae* is an excellent host for the heterologous production of fungal natural products, and has the potential to become an efficient chassis for the production of basidiomycete secondary metabolites in synthetic biology.

**Graphical Abstract:**

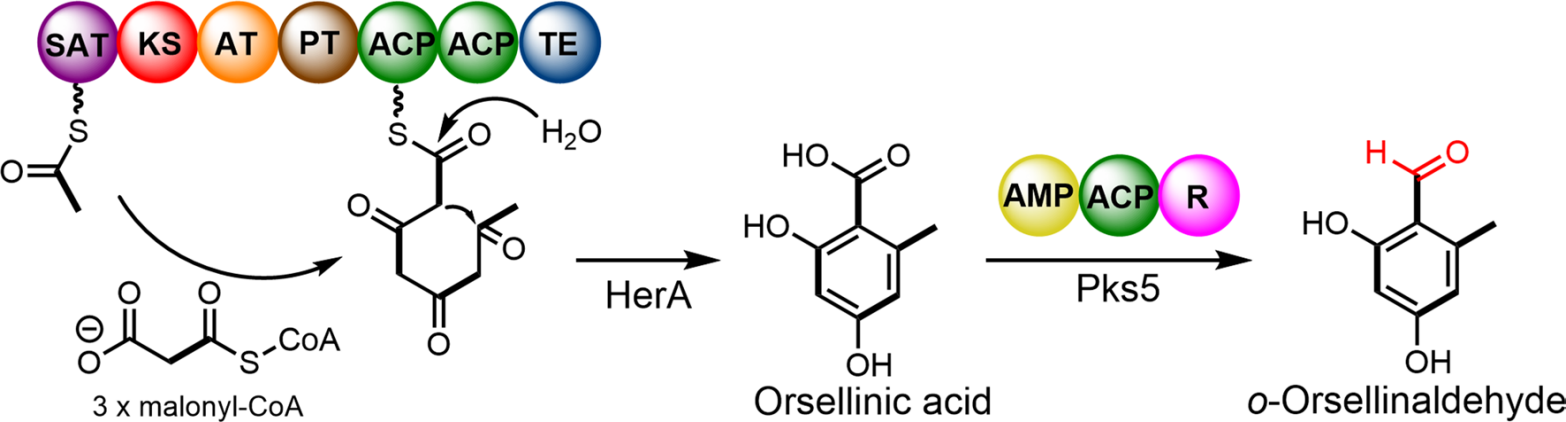

**Supplementary Information:**

The online version contains supplementary material available at 10.1186/s12934-023-02071-9.

## Background

**Fig. 1:**
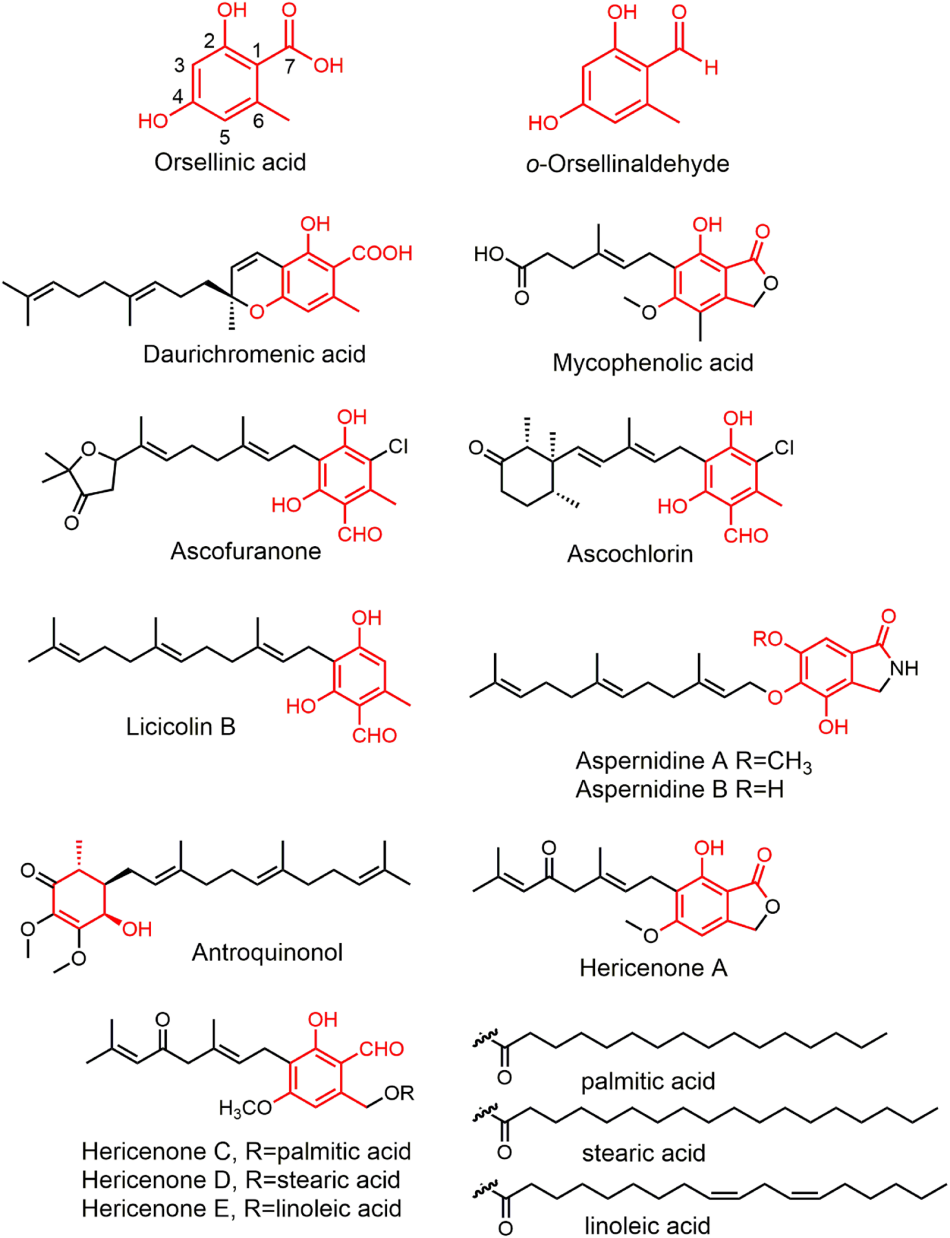
Representative natural products containing the OA scaffold

Orsellinic acid (OA) is a dihydroxybenzoic acid derivative with an extra methyl group, and its structural analogue *o*-Orsellinaldehyde is a benzene carbaldehydes compound. Their natural derivatives are typically contained isopentenyl units. More than 200 OA derivatives compounds have been isolated and identified in plants, lichens, fungi, and bacteria [1], which exhibit essential biological activities. For example, Daurichromenic acid (DCA), isolated from the leaves of the plant *Rhododendron dauricum*, has potent anti-HIV activity [2]. Mycophenolic acid (MPA), derived from the filamentous fungi *Penicillium brevicompactum*, has been a first-line immunosuppressive drug for organ transplantations and autoimmune diseases [3]. Ascofuranone (AF) and ascochlorin (AC) are produced by *Acremonium egyptiacum*, of which AF is a promising drug candidate against African trypanosomiasis and a potential anticancer lead compound [4]. Aspernidine A and B two phthalaldehydes, containing an *O*-farnesyl moiety, metabolites of *Aspergillus nidulans*, exhibited moderate antiproliferative activities [5]. Llicicolin B (LL-Z1272β), a prenylated aryl aldehyde produced by various fungi, such as *Stachybotrys bisbyi*, it not only inhibits pathogenic microorganisms and African trypanosomes, but is also less harmful to human cells [6, 7]. Antroquinonol, from the basidiomycete fungus *Antrodia camphorata*, has non-small cell cancer inhibitory activity and is currently a Phase II clinical lead drug [8]. Hericenone A, C, D, and E, isolated from the basidiomycete *H. erinaceus*, they were the first compounds discovered to have Nerve Growth Factor promoting activity and were pioneering drugs against Alzheimer's disease [9–11] (Fig. 1).

OA is a structurally simple aromatic polyketide formed by the stepwise condensation of acetyl coenzyme A with three malonyl coenzymes A. In fungi and bacteria, OA is mediated by repetitive type I PKS enzymes [12–14], while it is produced in plants by type III PKS [15]. In 2012, Ishiuchi et al. identified a PKS (CC1G_05377) from the model basidiomycete *Coprinopsis cinerea*, whose heterologous expression this gene in *Saccharomyces cerevisiae* produced OA [16]. In 2013, Lackner et al*.* identified *armB* in *Armillaria mellea* through genome mining. In vitro catalytic realization indicated that ArmB catalyzed acetyl-CoA and malonyl-CoA to produce OA [17]. PKS1 and 2 were identified from *Stereum* sp. when expressed in *A. niger* the OA was detected [18]. PKS63787 from *A. cinnamomea* and the metabolites of *Δpks63787* transformants were deficient in several aromatic compounds, including OA [19]. Although OA is the structural backbone of numerous secondary metabolites, only a few of the OA synthases described above have been characterized from basidiomycetes.*o*-Orsellinaldehyde is a natural product of reducing the C1-position carboxyl group of OA to an aldehyde group. There are two forms of enzymatic reactions that catalyze the conversion of OA to *o*-Orsellinaldehyde, one is the catalytic reaction responsible for the Non-ribosomal peptide synthase-like (NRPS-like) enzymes, such as StbB, AscB, ATEG_03630 [4, 7, 20], and the other is the catalytic reaction responsible for the R domain of PKS, such as PkfA, TropA [21, 22] (Additional file 1: Fig. S1). In 2019, Reyes-Fernández et al. identified Pks5 from the *U. maydis* genome, and based on gene knockout experiments, proved that the function of the enzyme is to convert OA into *o*-Orsellinaldehyde [23].

Currently, most OA derivatives are of plant origin [24], but they are present in relatively small amounts and difficult to isolate and extract. In recent years, the emergence of synthetic biology has promoted the development of related technologies. Through the concept and technology of synthetic biology, the construction of microbial cell factories not only dramatically reduces the production cost, but also provides a new way to protect rare plant resources and drugs development. *E. coli* and several *streptomyces* species are frequently utilized for bacterial genes. The most popular plant heterologous host for plant genes is the tobacco “*Nicotiana benthamiana*”, and the microbial chassis for these organisms is *Saccharomyces cerevisiae*. For fungal genes, there are several strains available, including *S. cerevisiae* and well-characterized *Aspergillus* sp. [25]. To achieves efficient biopreparation of OA and its derivatives, the filamentous fungus *A. oryzae* was selected as the heterologous expression host, which can synthesize polyketides, terpenoids, non-ribosomal peptides, and their post-modification products [26–28].

Through sequence similarity networks (SNNs) analysis, we found a gene *herA* in the genome of the basidiomycete *H. erinaceus*, that prediction involved in the synthesis of OA. Using *A. oryzae* as host, construction of *herA* heterologous expression strain (AO-*herA*), we not only verified the function of HerA, but also obtained an engineering strain of *A. oryzae* with high OA production. Next, transformed *pks5* from *U. maydis* into AO-*herA* transformants, and successfully obtained a strain-producing *o*-Orsellinaldehyde. Through the optimization of carbon sources and the comparison of fermentation methods, we finally determined the best production conditions for high-yielding OA and *o*-Orsellinaldehyde. This study provides a new strategy for the construction and optimization of the biosynthetic pathway of OA derivatives. In addition, our study also provides an effective method for the efficient expression of genes in basidiomycetes.

## Results and discussion

### Genome mining polyketide synthase in *H. erinaceus*

**Fig. 2:**
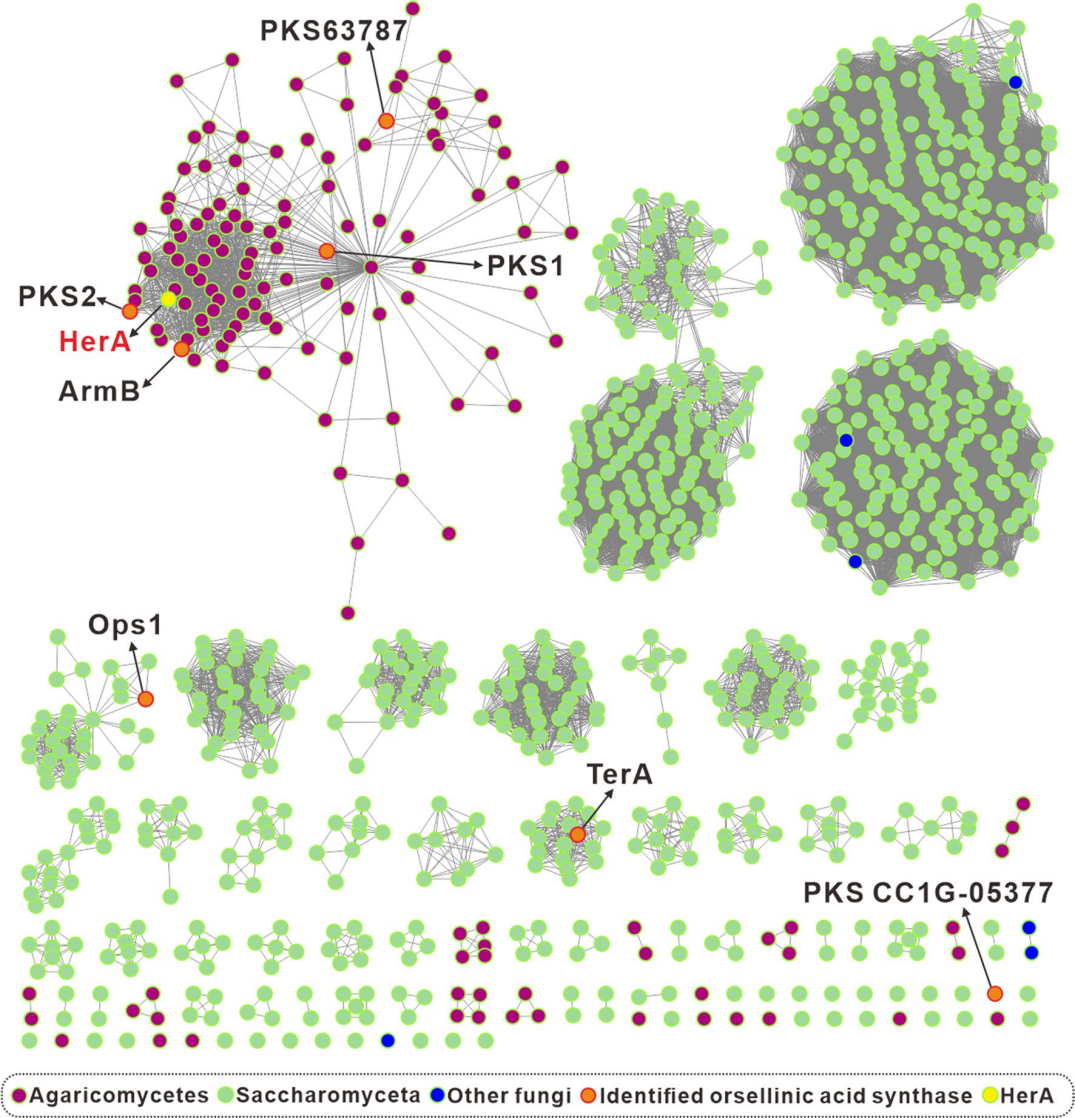
The SSNs network analysis based on ArmB and its homologous sequences. All homologous sequences were from Uniport and other databases. The purple group is from Basidiomycetes. The green group is from ascomycetes. The blue group is from other fungi. The orange dots represent identified OAS. The yellow dots represent HerA

OA is biosynthesized by non-reducing polyketide synthase (NR-PKS) [29]. ArmB, an orsellinic acid synthase (OAS) from *A. mellea*, has the canonical nonreducing architecture (SAT–KS–AT–PT–ACP–TE) [17]. The OAS gene from the basidiomycetes fungi has rarely been studied or identified. To obtain insights into the OAS homolog in the basidiomycetes fungi, a BLAST search of the fungi genomic database using ArmB as the target gene. For better visualization and classification, the SSNs were built for the 1000 curated NR-PKS using the Enzyme Function Initiative-Enzyme Similarity Tool [30]. The results showed that most OAS are derived from ascomycetes, and only a minority originates from basidiomycetes (Fig. 2). The reason for is that the number of ascomycetes in the reported genome database is much higher than that of basidiomycetes. Based on the data analyzed, all the reported genomes of basidiomycetes contain sequences with high homology for OAS, and this result indicates that it is ubiquitous in basidiomycetes. Therefore, identifying the functions of OAS in basidiomycetes, for the biosynthetic study of OA derivatives was essential.

*Hericium erinaceus*, also known as lion’s mane mushroom, is a widely distributed edible and medicinal fungus in Asian countries. The *H. erinaceus* mushroom contains a class of compounds “hericenones”, that promote the synthesis of Nerve Growth Factors [31]. This class of compounds is based on the OA backbone, the biosynthetic of hericenones processes remain unknown. Elucidation of the function of OAS in *H. erinaceus* will provide an insight into the biosynthesis of hericenones. Through SSNs analysis, we found an NR-PKS in the basidiomycete *H. erinaceus* and named it HerA. Through further analysis, HerA contained 2147 amino acid sequence, which was distributed in the same classic as the previously reported ArmB [17], CC1G-05377 [16], PKS1, 2 [18], and PKS63787 [19] from basidiomycetes, while Osp1 and TerA of ascomycete origin were in a different classic. According to bioinformatic analysis, except for PKS CC1G-05377, these enzymes share the structural SAT, KS, AT, PT, ACP, and TE domains for OA biosynthesis (Additional file 1: Table S1). Noteworthy, HerA contains two ACP domains. We speculated that it can synthesize OA.

### Heterologous expression of *herA* in *A. oryzae* to form OA

**Fig. 3:**
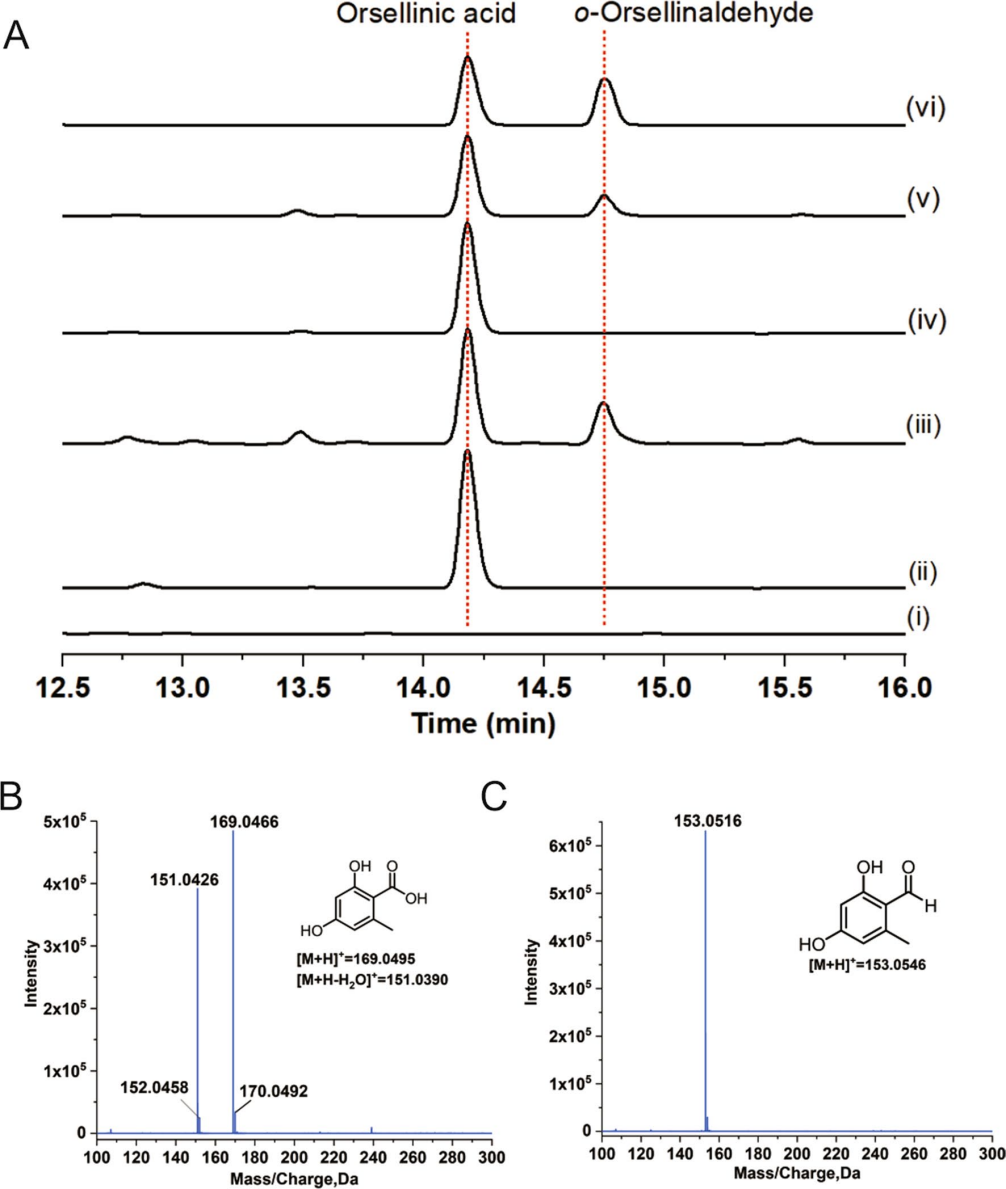
Heterologous expression and characterization of metabolites. **A** HPLC profiles of OA and *o*-Orsellinaldehyde produced by the transformants. (i) *A. oryzae* NSAR1, (ii) AO-*herA*, (iii) AO-*herA*-*pks5*, (iv) Bioconversion of OA by* A. oryzae* NSAR1, (v) Bioconversion of OA by AO-*pks5*, (vi) Standard of Orsellinic acid and *o*-Orsellinaldehyde. **B** MS spectrum of OA. **C** MS spectrum of *o*-Orsellinaldehyde

To investigate the function of HerA, the *herA* gene was amplified from the *H. erinaceus* CS4 genomic DNA (gDNA) and cloned into the pUARA2 plasmid. The pUARA2 containing *herA* was subsequently transformed into *A. oryzae* NSAR1 to obtain AO-*herA* transformants. HPLC analysis of the AO-*herA* mycelial extract showed a new peak with maximum UV absorption of 207, 262, and 300 nm (Additional file 1: Fig. S2), while this peak was not found in the control of the wild-type strain (Fig. 3A). To determine the structure of this compound, the AO-*herA* transformants were cultured on rice medium on a large scale. The crude ethyl acetate extract of the ferment was isolated by silica gel column chromatography and HPLC purification to obtain the pure monomeric compound. High-resolution electrospray mass spectrometry (HR-ESI–MS) analysis determined the molecular weight [M + H]^+^ 169.0466 of the compound with the presumed molecular formula of C_8_H_8_O_4_ (calculated as [M + H]^+^169.0495) (Fig. 3B). Further NMR examination characterized the structure, and its NMR data (Additional file 1: Figs. S3, S4, and Table S2) were consistent with the reported NMR data of OA [32].

The heterologous expression of *herA* in *A. oryzae*, that indicates HerA is an OAS from the *H. erinaceus* and is responsible for OA production. The ability of *A. oryzae* to correctly recognize the seven introns of *herA* (Additional file 1: Table S3), as a filamentous fungus, it can efficiently express the gDNA gene of basidiomycetes.

### Functional analysis of Pks5

*Ustilago maydis* is a typical plant pathogenic fungus, and there are five PKS genes in its genome sequence. A recent study showed that *pks5* is a polyketide synthase-encoding gene from *U. maydis* associated with the biosynthesis of melanin. Inactivation of *pks5* results in the loss of *o*-Orsellinaldehyde, and the accumulation of OA in *U. maydis*. This result suggests that the function of Pks5 is responsible for the conversion of OA to *o*-Orsellinaldehyde [23]. Bioinformatics analysis revealed that Pks5 contains two introns, and three structural domains, AMP, ACP, and R (Additional file 1: Table S1), with 30% identity to the NRPS-like enzyme ATEG_03630. To construct a cell factory to produce *o*-Orsellinaldehyde, we cloned the *pks5* gene from *U. maydis* and transferred it into the AO-*herA* strain to obtain an AO-*herA*-*pks5* transformant. HPLC analysis revealed a new peak in the metabolites of the AO-*herA*-*pks5* transformants relative to the AO-*herA* strain, whose retention time was consistent with that of the standard *o*-Orsellinaldehyde (Fig. 3A). To further confirm the function of *pks5*, an *A. oryzae* transformant containing *pks5* was constructed. AO-*pks5* was fed with OA as substrate, and HPLC analysis showed that the AO-*pks5* strain could convert OA into a new compound with a retention time consistent with that of *o*-Orsellinaldehyde (Fig. 3A). HR-ESI–MS analysis showed that the compound had a [M + H]^+^ of 153.0516, with the presumed molecular formula C_8_H_7_O_3_ (calculated as [M + H]^+^153.0546) (Fig. 3C). The ^1^H and ^13^C NMR data of this compound (Additional file 1: Figs. S5, S6, and Table S2) agree with the known o-*Orsellinaldehyde* data [30]. Our results indicated that *A. oryzae* can correctly splice the two introns of *pks5* and that the incomplete structural domain with Pks5 has the function of converting carboxyl groups to aldehyde groups.

### Optimization of OA and *o*-Orsellinaldehyde yields

**Fig. 4:**
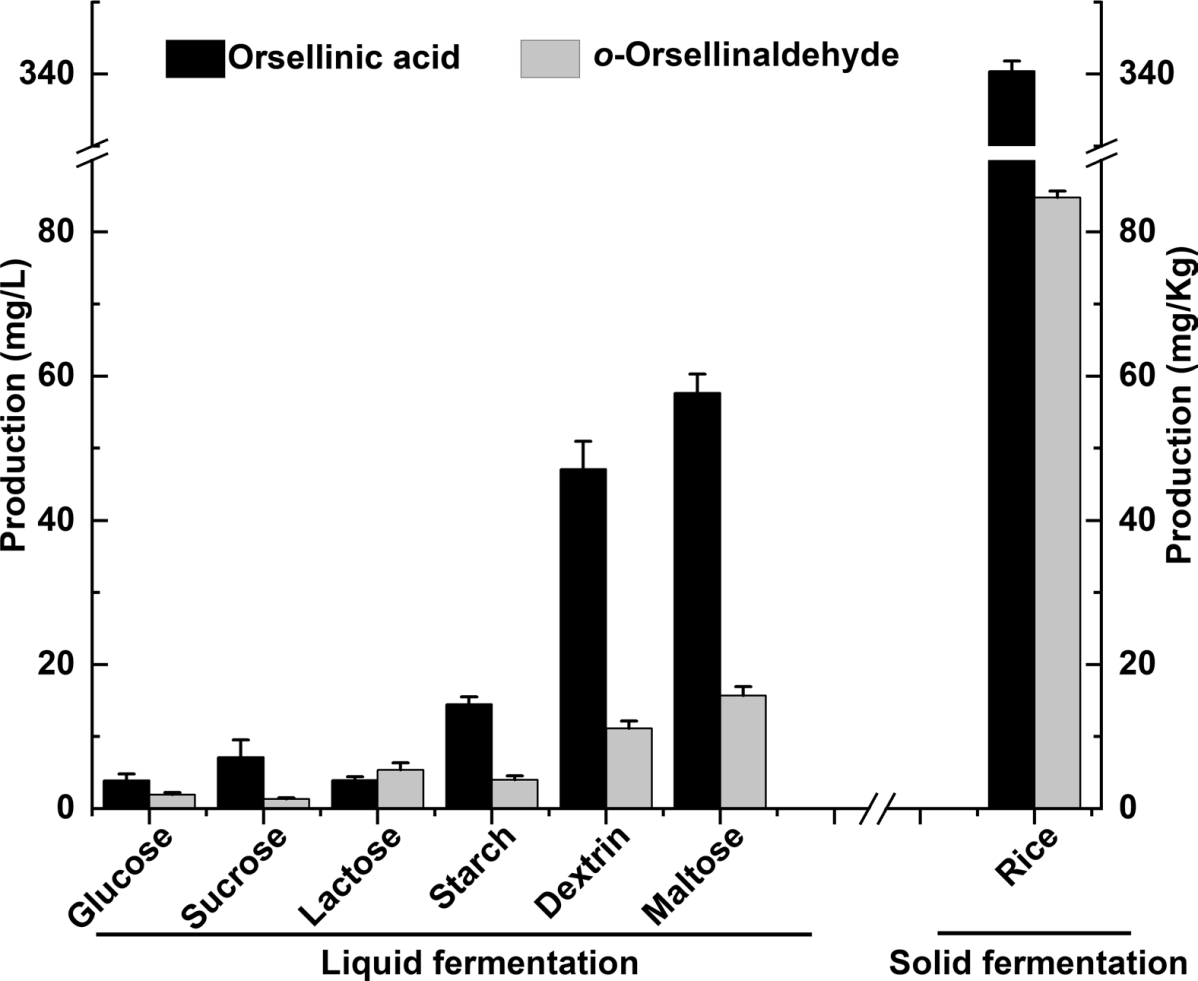
Effects of different carbon sources and fermentation conditions on the yield of Orsellinic acid and *o*-Orsellinaldehyde

Since both OA and *o*-Orsellinaldehyde are important pharmaceutical intermediates and chemical raw materials, we proceeded to optimize the yields of the two compound-producing. Carbon sources provide the energy for microbial growth, and the carbon skeleton for metabolites. Considering the cost of large-scale production, we needed to screen for carbon sources that were less expensive and high yields of metabolites. The six different carbon sources (glucose, sucrose, lactose, dextrin, maltose, and starch) were selected for carbon source screening to compare the effects on the production of OA and *o*-Orsellinaldehyde. The results showed that the addition of different carbon sources had different effects on OA and *o*-Orsellinaldehyde production, where the highest OA and *o*-Orsellinaldehyde yields of 57.68 mg/L and 15.71 mg/L, respectively, were obtained when maltose was used as the carbon source (Fig. 4). In addition, OA and* o*-Orsellinaldehyde production was higher than that of glucose, sucrose, and lactose when starch and dextrin were used as carbon sources. In the present experiment, the *α*-amylase promoter was used for the expression of exogenous genes, and maltose was able to induce the expression of amylases, including *α*-amylase in *A. oryzae* [33]. Therefore, the yield of OA and *o*-Orsellinaldehyde was highest when maltose was used as the carbon source.

Compared to liquid cultures, solid cultures with rice medium showed a 5.90- and 5.40-fold increase in OA and *o*-Orsellinaldehyde production, with yields of 340.41 mg/kg and 84.79 mg/kg, respectively (Fig. 4). Tagami K et al. reconstructed the biosynthesis of the indole diterpenoid in *A. oryzae*, when using maltose as a carbon source for liquid fermentation, the yield of paxilline was 35 mg/L [34], however the yield of aflatrem was 54 mg/Kg when rice was used as a solid medium [35]. This suggests that during solid culture, *A. oryzae* has support attached to it during growth. It can form a differentiation between trophic and aerial mycelium, this form facilitates the production of a large number of enzymes, that able to increase the efficiency of heterologous expression of proteins, thus increasing the yield of metabolites. In contrast, under liquid culture conditions, which are relatively unstable, *A. oryzae* tends to form balls of varying sizes, which may have affected the production of metabolites. Furthermore, in the later stages of liquid culture, the pH of the medium tends to be acidic. Aldehyde groups are unstable under acid conditions. This may be one of the reasons why *o*-Orsellinaldehyde is more productive in solid medium. So far, *A. oryzae* is suitable for heterologous expression of basidiomycetes PKS products.

Here, we have demonstrated the successful expression of PKS genes *herA* and *pks5* from basidiomycetes in *A. oryzae*, resulting in the construction of a cell factory capable of producing OA and *o*-Orsellinaldehyde. Our findings highlight the potential of *A. oryzae* as a promising platform to produce mushroom polyketide compounds, which offering several advantages such as low cost, high efficiency, environmental friendliness, and sustainability. These results not only provide valuable insights into the biosynthesis of important pharmaceutical intermediates, but also offer an important contribution to the elucidation of gene function.

## Conclusion

In this work, we identified the functions of HerA and Pks5 by heterologous expression in *A. oryzae* as hosts, and simultaneously obtained OA and *o*-Orsellinaldehyde producer strains. Carbon source optimization and screening of fermentation methods pointed to the optimal production of OA and* o*-Orsellinaldehyde. This result lays the foundation for the possible future industrial production of both compounds.

It is well known that basidiomycetes are a demanding species with different growth cycles. In some cases, the correct cDNA sequence cannot be obtained. This limits the research on the biosynthesis of basidiomycetes natural products. *A. oryzae* is an excellent host for heterologous expression. It is competent to directly express intron-containing genes, with the ability to correctly splice mRNA and translate. This strategy saves time in basidiomycetes cultivation and provides a new approach to the biosynthesis of basidiomycetes natural products. We believe that *A. oryzae* heterologous expression tools will have more widespread applications.

## Material and methods

### General experimental procedures

All reagents commercially supplied were used as received. HPLC analysis was performed using an Agilent 1260 Series with a DAD detector (California, CA, USA). ^1^H- and ^13^C-NMR spectra were recorded on Bruker AVAN CEIII HD 500. Chemical shifts were reported as δ scale in ppm as an internal reference (CD_3_OD; ^1^H NMR = 3.31 ppm, ^13^C NMR = 49.0 ppm). Mass spectra were obtained with an AB SCEIX Triple TOF 6600. Column chromatography was carried out on C18 silica gel (Agilent Technologies. USA). Oligonucleotides for polymerase chain reaction (PCR) were purchased from RuiBiotech Biotechnology Co., Ltd.

### Strains and culture conditions

*H. erinaceus* CS-4 (CCTCC AF 2018025) [36] was cultivated at 25 °C, 170 rpm in potato dextrose broth (PDB) liquid medium for one week and used as a source for the cloning of *herA* gene. The *U. maydis* (CGMCC 5.208) was grown at 28 °C, 200 rpm in YEPS (yeast extract-pep-tone-sucrose: 1% yeast extract, 2% peptone, 2% sucrose, 100 mL) for one week and used as a source for the cloning of *Pks5* gene. Standard DNA engineering was performed with *Escherichia coli* DH5α and culture at 37 ℃ for grown. *A. oryzae* NSAR1 (*niaD-, sC-, ΔargB, adeA-*) was used as the fungal heterologous expression host in this study, and growth at 30 °C, 200 rpm in DPY (dextrin-polypeptone-yeast extract: 2% dextrin, 1% polypeptone, 0.5% yeast extract, 100 mL) medium supplemented with appropriate nutrients.

### Extraction of the genomes and construction of plasmids

Extraction of the genomes of *H. erinaceus* and *U. maydis* was carried out according to the literature procedure [34]. The *herA* was divided into two fragments for amplification from the genome of *H. erinaceus* with the primers described in Additional file 1: Table S4, and then introduced into pUARA2 vector using ClonExpress MultiS One Step Cloning Kit (Vazymebiotech Laboratories) to construct expression plasmids, pUARA2-*herA* (Additional file 1: Table S5). The *pks5* was amplified from the genome of *U. maydis* with the primers described in Additional file 1: Table S4 and then introduced into pUSA2 vector, using a ClonExpress Ultra One Step Cloning Kit (Vazyme Biotech Co., Ltd) to construct expression plasmids, pUSA2-*pks5* (Additional file 1: Table S5).

### Transformation of *Aspergillus oryzae*

Transformation of *A. oryzae* NSAR1 [37] was carried out by the previously reported protoplast–polyethylene glycol method [38]. The plasmid pUARA2-*herA* was used for the first transformation to construct AO-*herA*. This transformant was further transformed with pUSA2-*pks5* to construct AO-*herA-pks5*. pUSA2-*pks5* was used for the transformation to construct AO-*pks5*.

### Biotransformation of OA using AO-*pks5*

Mycelia of the transformant with *pks5* were inoculated into MPY (maltose-peptone-yeast extract: 3% maltose, 1% polypeptone, 0.5% yeast extract) medium (2 mL) containing appropriate nutrients in 10 mL test tube. OA (20 μg, methanol solution) was then administered to the culture medium. After an additional 3 days of incubation at 30 °C, 200 rpm. The mycelium was removed by filtration, and the broth was extracted with ethyl acetate, then the organic layers were concentrated in vacuo. The crude extracts were directly analyzed by HPLC and LC–MS.

### Extraction and analysis of metabolites

Mycelia of AO*-herA* and AO*-herA-pks5* transformants were inoculated into a solid medium containing polished rice (1 g) and appropriate adenine at 30 °C for 10 days. After extraction with ethyl acetate, the extract was concentrated in vacuo to afford crude extracts. The crude extracts were analyzed by HPLC equipped with an Agilent TC-C18 (250 mm × 4.6 mm) at the following conditions: 0–5 min, 10% B; 5–20 min, a linear gradient 10–100% B; 20–30 min, 100% B (A: H_2_O + 0.1% of formic acid, B: CH_3_OH + 0.1% of formic acid) at a flow rate of 1 mL/min. Samples were analyzed using a TripleTOF 6600 mass spectrometer (AB/SCIEX, Milford, MA) and an HPLC system (AB/SCIEX). Chromato- graphic separation was achieved using a 150 mm × 4.6 mm, 2.6 μm Kinetex C18 100A column (Phenomenex) at the following conditions: 0-10 min, 5–100% B;10–15 min, 100% B (A: H_2_O + 0.1% of formic acid, B: CH_3_CN + 0.1% of formic acid) at a flow rate of 0.6 mL/min.

### Effect of different carbon sources on the production of OA and *o*-Orsellinaldehyde

The spore suspension (1 × 10^8^ Cell/mL) of AO-*herA-pks5* transformant was inoculated into 100 mL of PY (1% polypeptone, 0.5% yeast extract) liquid medium supplemented with 2% with different carbon sources (glucose, sucrose, lactose, dextrin, starch, and maltose) in 500 mL Erlenmeyer flasks. After 3 days of incubation at 30 ℃ and 200 rpm. The mycelium was removed by filtration, and the broth was extracted with ethyl acetate three times. Then, the organic layers were concentrated in vacuo. The crude extracts were obtained and analyzed by the above method. All experiments were replicated three times.

### Isolation and purification of each metabolite

Mycelia of AO-*herA*-*pks5* transformants were inoculated into a solid medium containing polished rice (1 kg) and appropriate adenine at 30 °C for 10 days. The mycelia were extracted with ethyl acetate at room temperature overnight. The ethyl acetate layer was washed with brine and concentrated in vacuo. The crude extracts were isolated using silica gel column chromatography (Petroleum ether: ethyl acetate, 6:1 to 2:1). The isolated compounds were further purified by semi-preparative HPLC.

## Supplementary Information

**Additional file 1:** **Figure S1.** NR-PKS and NRPS-like enzyme has been reported to be responsible for the formation of synthetic aldehyde groups. **Figure S2.** UV spectroscopy of OA. **Figure S3.** ^1^H-NMR spectrum of OA (CD_3_OD-*d*4, 500 MHz). **Figure S4.** ^13^C-NMR spectrum of OA (CD_3_OD-*d*4, 125 MHz). **Figure S5.** ^1^H-NMR spectrum of *o*-Orsellinaldehyde (CD_3_OD-*d*4, 500 MHz). **Figure S6.** ^13^C-NMR spectrum of *o*-Orsellinaldehyde (CD_3_OD-*d*4, 125 MHz). **Table S1.** Domain organizations of PKS in this study. **Table S2.** NMR data for OA and *o*-Orsellinaldehyde in CD_3_OD-*d*4 (500 MHz for ^1^H NMR,125 MHz for ^13^C NMR). **Table S3.** DNA and protein sequences. **Table S4.** Primers used in this study. **Table S5.** Plasmids constructed in this study.

## Data Availability

All data for this study are included in this published article and its additional file.

## Abbreviations

OA: Orsellinic acid
PKS: Polyketide synthase
NRPS-like: Non-ribosomal peptide synthase-like
SSNs: Sequence similarity networks
AO: *Aspergillus oryzae*
NR-PKS: Non-reducing polyketide synthase
OAS: Orsellinic acid synthase
HPLC: High performance liquid chromatography
HR-ESI–MS: High resolution electrospray ionization mass spectrum
NMR: Nuclear Magnetic Resonance
gDNA: Genomic DNA
