# Supplementary material for: High-efficient production of mushroom polyketide compounds in a platform host Aspergillus oryzae
Source: Microb Cell Fact. 2023 Mar 30;22:60. doi: 10.1186/s12934-023-02071-9 (PMC10064546; doi:10.1186/s12934-023-02071-9)
Supplement: Supplementary file 1 — Additional file 1: Figure S1. NR-PKS and NRPS-like enzyme has been reported to be responsible for the formation of synthetic aldehyde groups. Figure S2. UV spectroscopy of OA. Figure S3. 1H-NMR spectrum of OA (CD3OD-d4, 500 MHz). Figure S4. 13C-NMR spectrum of OA (CD3OD-d4, 125 MHz). Figure S5. 1H-NMR spectrum of o-Orsellinaldehyde (CD3OD-d4, 500 MHz). Figure S6. 13C-NMR spectrum of o-Orsellinaldehyde (CD3OD-d4, 125 MHz). Table S1. Domain organizations of PKS in this study. Table S2. NMR data for OA and o-Orsellinaldehyde in CD3OD-d4 (500 MHz for 1H NMR,125 MHz for 13C NMR). Table S3. DNA and protein sequences. Table S4. Primers used in this study. Table S5. Plasmids constructed in this study. [file 12934_2023_2071_MOESM1_ESM.docx]

**Supporting Information**

**High-efficient production of mushroom polyketide compounds in a platform host *Aspergillus oryzae***

Haiyan Han^1^, Chunyan Yu^1^, Jianzhao Qi^1^, Pengchao Wang^1^, Peipei Zhao^2^ Wenbing Gong^3^, Chunliang Xie^3^, Xuekui Xia^2^*, Chengwei Liu^1^*

1. Key Laboratory for Enzyme and Enzyme-like Material Engineering of Heilongjiang, College of Life Science, Northeast Forestry University, Harbin, 150040, Heilongjiang, China.

2. Biology Institute, Qilu University of Technology (Shandong Academy of Sciences), Jinan, 250103, Shandong, China.

3. Institute of Bast Fiber Crops, Chinese Academy of Agricultural Sciences, Changsha, 410205, Hunan, China.

* Corresponding authors:

Xuekui Xia, Biology Institute, Qilu University of Technology (Shandong Academy of Sciences), Jinan, 250103, Shandong, China.

TEL: +86-531-82605332, E-mail: [xiaxk@sdas.org](mailto:xiaxk@sdas.org);

Chengwei Liu, Key Laboratory for Enzyme and Enzyme-like Material Engineering of Heilongjiang, College of Life Science, Northeast Forestry University, Harbin, 150040, Heilongjiang, China.

Tel: +86-451-82191378, E-mail: [liuchw@nefu.edu.cn](mailto:liuchw@nefu.edu.cn).

**Table of Contents**

**Supplementary experimental section** **3**

**Supporting Figures** **4**

Figure S1. NR-PKS and NRPS-like enzyme has been reported to be responsible for the formation of synthetic aldehyde groups. 4

Figure S2. UV spectroscopy of OA. 4

Figure S3. ^1^H-NMR spectrum of OA (CD_3_OD-*d*4, 500 MHz). 5

Figure S4. ^13^C-NMR spectrum of OA (CD_3_OD-*d*4, 125 MHz). 5

Figure S5. ^1^H-NMR spectrum of *o*-Orsellinaldehyde (CD_3_OD-*d*4, 500 MHz). 6

Figure S6. ^13^C-NMR spectrum of *o*-Orsellinaldehyde (CD_3_OD-*d*4, 125 MHz). 6

Supporting Tables:

Table S1. Domain organizations of PKS in this study. 7

Table S2. NMR data for OA and *o*-Orsellinaldehyde in CD_3_OD-*d*4 (500 MHz for ^1^H NMR ,125 MHz for ^13^C NMR). 8

Table S3. DNA and protein sequences. 9

Table S4. Primers used in this study. 13

Table S5. Plasmids constructed in this study. 13

**Supplementary experimental section**

**Sequence similarity network analysis**

SSN (https://efi.igb.illinois.edu/efi-est/) was used to analysis OA. The EFI-EST(https://efi.igb.illinois.edu/efi-est/) was used to generate SSNs. Filter type was set as E-Value, and filter value was set as 50. Considering that the length of the ArmB sequence is 2209 amino acids, the length of the homologous sequence is restricted to 1200-3000 amino acids. Sequence function space in the SSNs was analyzed with Cytoscape V3.9.1, a desktop platform for visualizing complex networks (http://www.cytoscape.org/), using node attributes from the UniProtKB and other databases to assist segregating the SSN into isofunctional clusters. Characterized OA of different species are highlighted with distinct colors.

**Supporting Figures:**


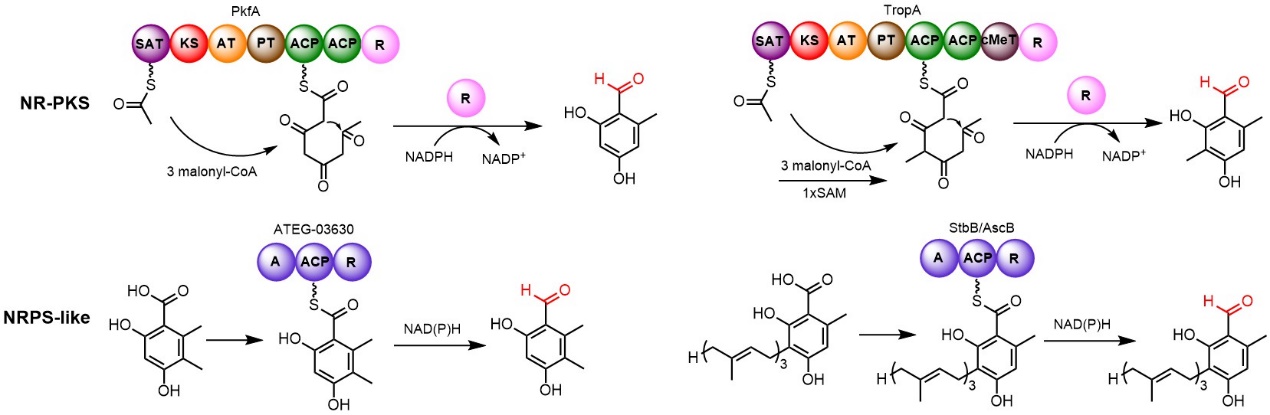


**Figure S1. NR-PKS and NRPS-like enzyme has been reported to be responsible for the formation of synthetic aldehyde groups.**

***

***

**Figure S2. UV spectroscopy of OA.**

**
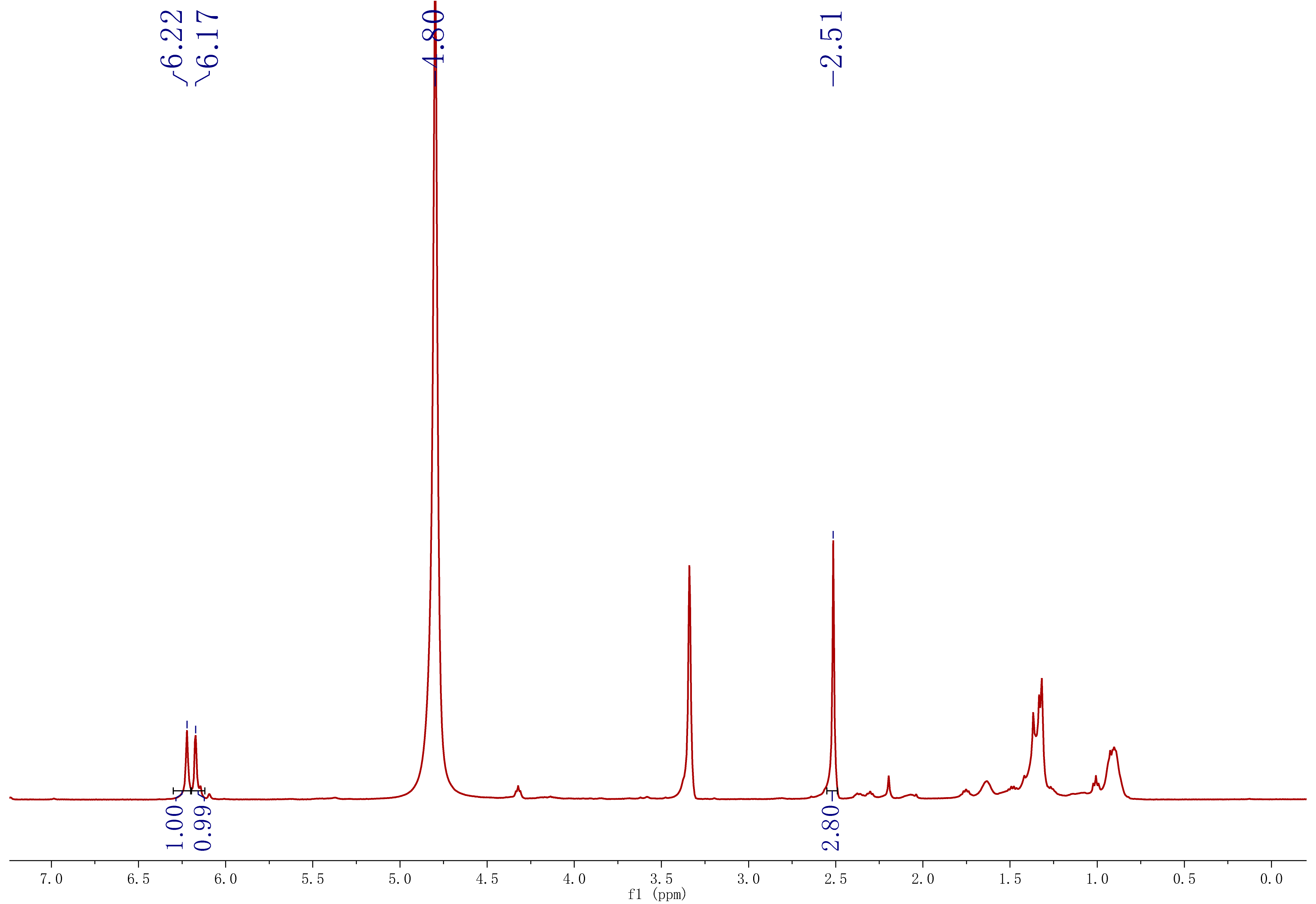
**

**Figure S3.** **^1^H-NMR spectrum of OA (CD_3_OD-*d*4, 500 MHz).**

**
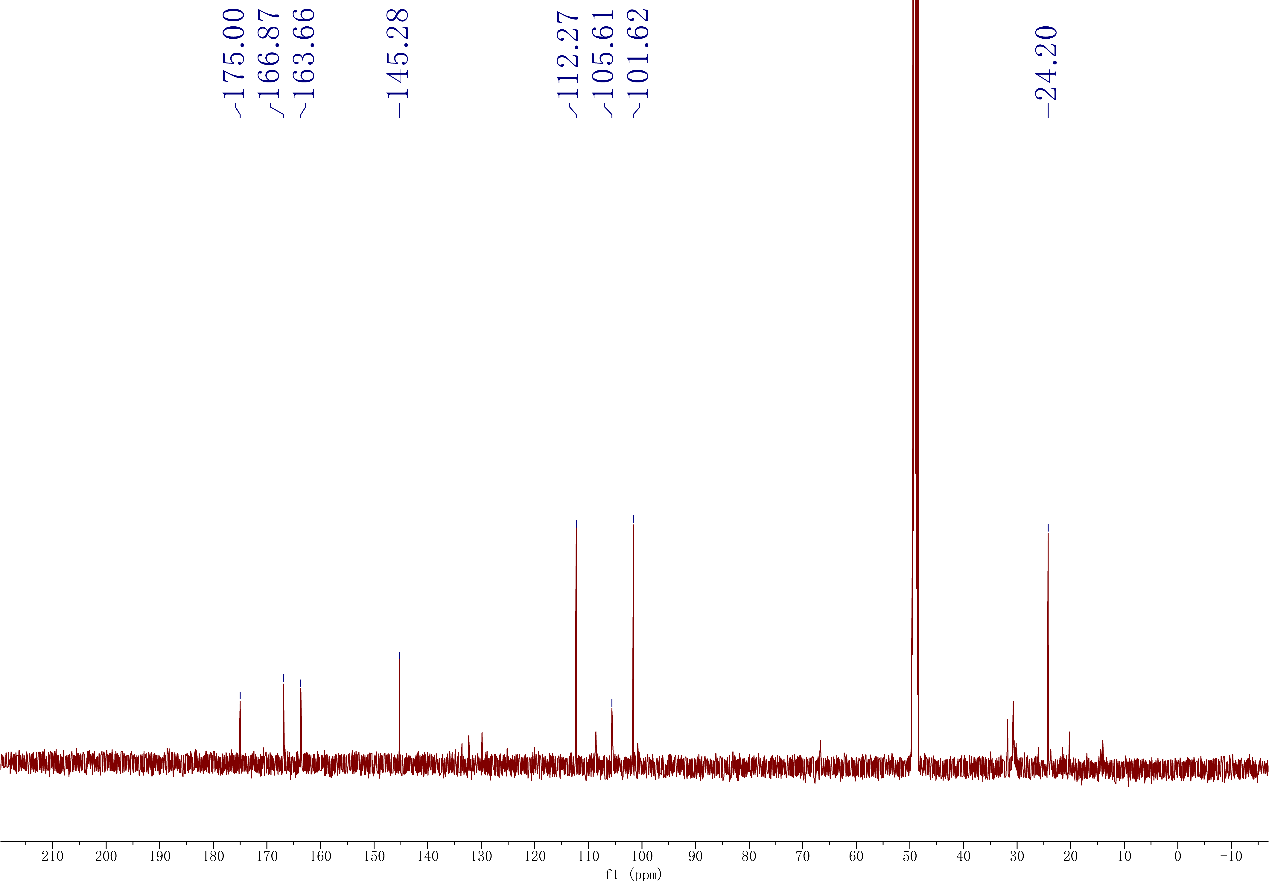
**

**Figure S4.** **^13^C-NMR spectrum of OA (CD_3_OD-*d*4, 125 MHz).**


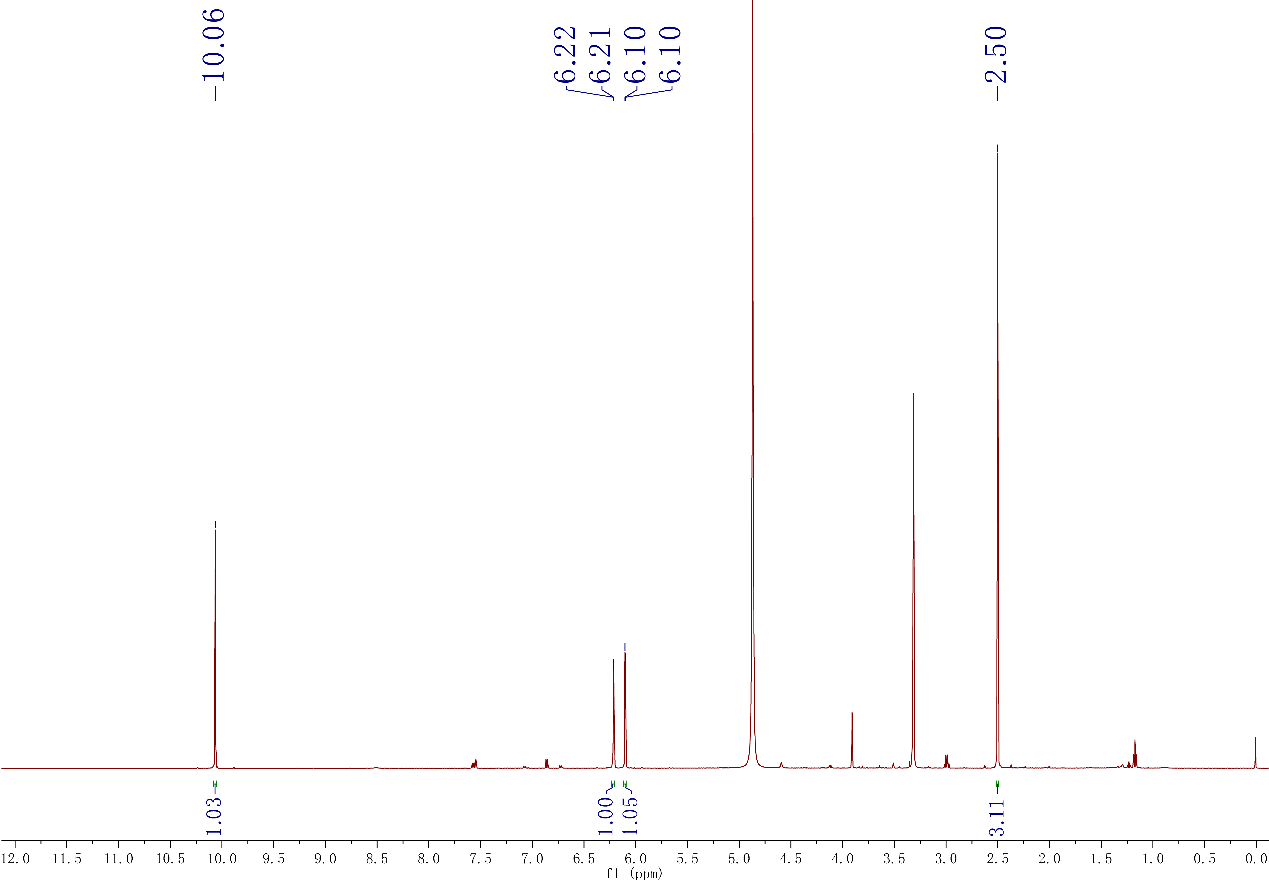


**Figure S5. ^1^H-NMR spectrum of *o*-Orsellinaldehyde (CD_3_OD-*d*4, 500 MHz).**

**
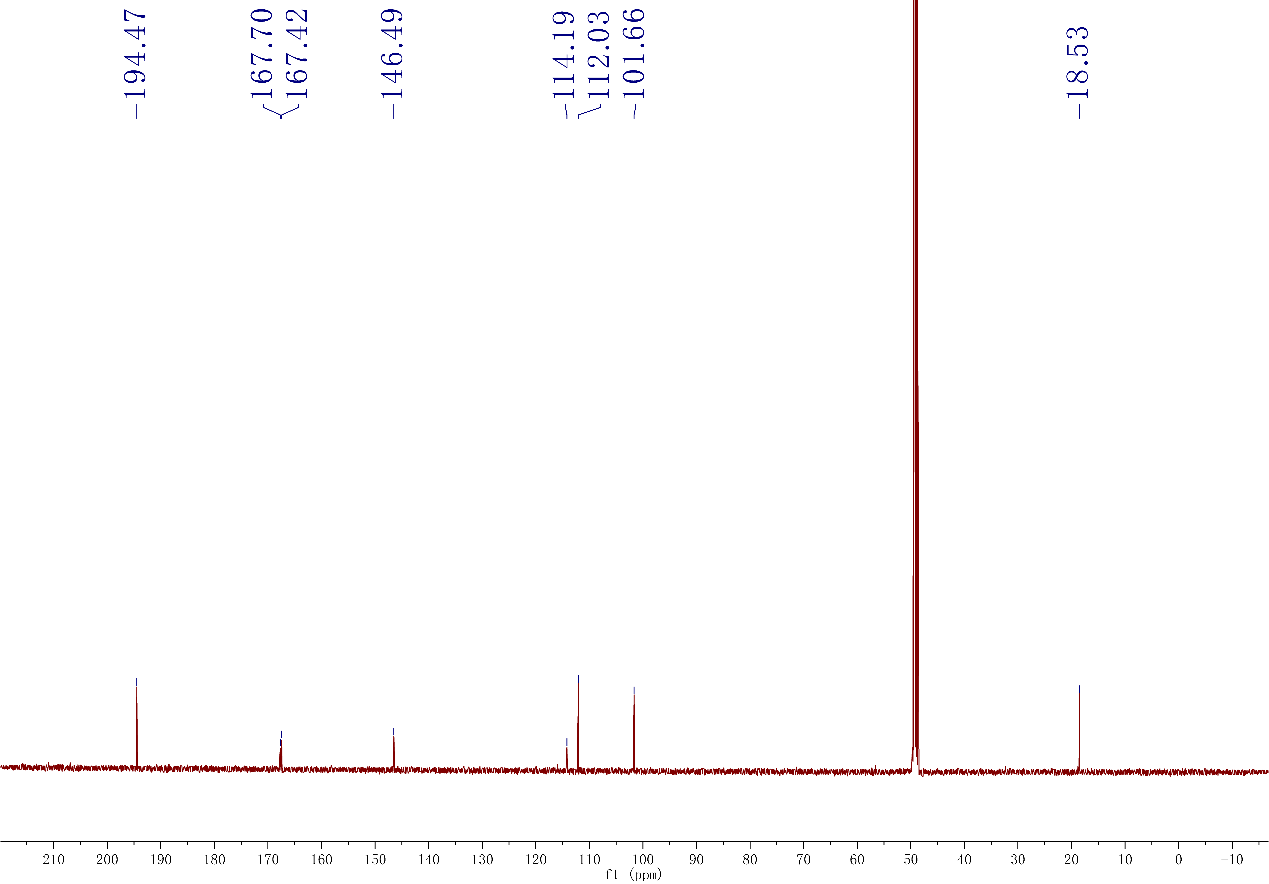
**

**Figure S6. ^13^C-NMR spectrum of *o*-Orsellinaldehyde (CD_3_OD-*d*4, 125 MHz).**

**Supporting Tables**

**Table S1. Domain organizations of PKS in this study. SAT: starter unit acyltransferase; KS: ketosynthase; AT: acyltransferase; PT: product template; ACP: acyl carrier protein; TE: thioesterase.**

| **Gene** | **Accession Number** | **Origin** | **Domain organization** |
| --- | --- | --- | --- |
| **HerA** | -- | *Hericium erinaceus* |  |
| **Pks5** | XP_011390603.1 | *Ustilago maydis* |  |
| **PKS (CC1G_05377)** | XP_001835415.2 | *Coprinopsis cinerea* |  |
| **ArmB** | I3ZNU9.1 | *Armillaria mellea* |  |
| **PKS1** | APH07629.1 | *Agaricomycetes* |  |
| **PKS2** | APH07628.1 | *Agaricomycetes* |  |
| **PKS63787** | KX683290.1 | *Antrodia cinnamomea* |  |
| **OpS1** | J4UHQ6.1 | *Beauveria bassiana* |  |
| **TerA** | Q0D1N9.1 | *Aspergillus terreus* |  |

**Table S2. NMR data for OA and *o*-Orsellinaldehyde in CD_3_OD-*d*4 (500 MHz for ^1^H NMR ,125 MHz for ^13^C NMR).**

|  | OA | | *o*-Orsellinaldehyde | |
| --- | --- | --- | --- | --- |
| position | *δ*_C_ | *δ*_H_ (*J* in Hz) | *δ*_C_ | *δ*_H_ (*J* in Hz) |
| 1 | 105.6, C |  | 114.2, C |  |
| 2 | 163.7, C |  | 167.4, C |  |
| 3 | 101.6, CH | 6.17, S | 101.7, CH | 6.10, d |
| 4 | 166.9, C |  | 167.7, C |  |
| 5 | 112.3, CH | 6.22, S | 112.0, CH | 6.21, d |
| 6 | 145.3, C |  | 146.5, C |  |
| 7 | 175.0, C |  | 194.5, CHO | 10.0, S |
| 6-CH_3_ | 24.2, CH_3_ | 2.50, S | 18.5, CH_3_ | 2.50, S |

**Table S3. DNA and protein sequences.** **Highlighted in yellow are the intron regions** **predicted in this study.**

| **Gene** | **DNA sequences** | **Predicted protein sequences** |
| --- | --- | --- |
| **HerA** | TCAAGCAGCAAGGCTCTCGAGGTGTGCGCACCCCTCAGCAATGCGACGAGAAACTTCCTCGATCTAAAAATCTATGGTCAGCGACAAGGCGTCTCAAGAGACAGCAGCAAGTACTTACATTAGAGGTGTGGAAAGGCTGGAAATGGTTGCCGGGGATGTCGATGACCTTGATCGGCGTGCCCACGACGCGCTCCCACCCGGATATGGCCAGCTGCGCATCGCTCCTGTCCGCAAGCCACGTCGGCACGTCGGCGACGCCGGCGGGTTTGAAGCCCTCTCTCGAGCGGAGGAGGACGATCGAGGGAAATGGCCCGCCCGCGGCAAGCGGGTCATAGCGGCCGAGCATGCGCGAGTTCATCTGGAACTGCGTCTTCACGAGCTTGCCGACCTGCGTGTCGGCGCGGCCATCCAGCTTCGCGACGGACTCGAGGAGGGCGTCGGAGAGCGGAACGTGGGCGAGAGGAGTAGGGGAGTCGATGAGGAGGACGCCCTTCACCGCAACGCCCTTCTTCATGAGCTGACACGCAGCTTCGAAGGCAACGACACCACCGAACGACCAGCCTGAGATAAACAATTATCAACAAAGCAAGAATAAGGGAGCGATGGAGGGTACTCGCCACCAAGGATAAGAGGCTCAGAGGTCGTCTTCGTCGCGAACTCAGAGTACTCCGCAGCCATCGACACAACACTCTCCCACGGCTGGCTGGTGATGAAGTGGGGATTATGAATGCCCCAGATGTCGCGGTCAAGCGGGGAGAGGCGCTGGATGTAGTTCACAAGGCCGCTACCATCGTGAATCAGGAATAAGGGCGCGCGGCCGGGTGTCTTCGCCTTCTGCGCACCGACGGGAACCGAGTCGAGGCGCAGCGCGGTGACGAGCGGGTTGAGGTTGCCGTCGACGATCTGCGCGACTTTCGCGGCGGTCGCGATGTCCGAGTCCTTGTAGTCCGAAGGGTGCACCTCGGTCTCCTTAACGGCCTCGACGGCCTTGCCCCGAGGACGCAGCTGCGATGTGAGGAAGGCGTTGACGGCCTTCGCCGTTGTGTATGTCTCGAACAGGGTGGTCGGCAGCGTCAGCGAGTACTCGCTCTGCAGGGCCGAATGCGCCTCGATGGATGTCAGCGAGTCTAAGCCGAGCGATTCGAAGTCCGCGTCGTCGGTGATCTCCTGGAGGCCGATGCCGAGGACGGACGCCAGCAGCGGCTTGACGTCGACCTCGTCGAAGTTCGCGATGACGCTGGGCTCGCCAAGCTTCTCGTCAGTGACGAGTGTGCGCGGCGTGGATGGGCCAGAGTCCTCCTGGGAGAACTTGGACGACACCTCAGCGACGATCTGGGCAACATTGCGGCACGAGGAAAGGACGTTCGCGTCAAGGTCAGCACTAGGGAAGGCGCTCTGCATCTTGGTGAAGATCTCAATGGACATCAGCGAATCCACGCCGTACGCCTCGAGGTCTCCGTCGGGCTGGATCGTGGATGCAGTGATGTCGCAAGTCTCGGCCACGATGCGCGTGACCTCCGCGAGGACGTCGATAGTAGGTGCTGTATCTCGAGGCGGCTCGACGAAGGTGATTGAGGAAACGACCTTGGAGGACATCGGGGAAGCAGGCGCAGCCTTCTTCACAGGCGCCTCCGCCCGCTTCGGCGCAGCATGCGCCGGGCTAGTGCCCGCGGCCATCGCGAGACCACGCTTCAGGCTATTGAGGCGCACCTTGCGGAAGTGCATGCCCTTGAGGTGCGCGACGATCTTACCTGGAGACTTCAACTGGACGGCGTAGGCCTCCGCAAGCATGACGCCCTCATCCGCGACCCAGGCGTTGCTGATCAGCACGCCGTACTCCGCGTCGTTGTCGATCAAAGCCGGGATCGCCTTCACTGTGTCGACCTTGCTGCAGATGTAGGCATCGTTGACTCCGCCCTGCATGTTCGCGACGAAGCCGGCAACGTGAAGCATGGTGTCCATGAAGACAGGGTGGACGATAAACTTGCTGCGGTCGTGATCGCGAGGCAGCTGGACGATCGCGTATGCTTCCATACCGTTCGAGGCGACGGTGAGCGTCTTCATCGTGTGGTACTCCTTTGCGTAGTCGACCACGCGCGGGAAGATGACCTCGTATGCCGTGCGAGTAGAGAACGTCTCCGCGAAACCATCGTTAGGCGACGAGACGGAGCTGATTTGACGAGTTACGATGGGAAGAACGCGAGCGAATTTGGTTGAGGCCTTCGATGTCGACTGGTGCTTGAACTTCCCGAAGCAATGGACCTCCTCGGGCGAGCCATCTACCTGCGAGCCAACGCTGAAAGAGCCCGAGCCATCAACGTCAAGTGTGATCGATGTCCTGACCATCCGGGCAACGTGCTCGTTGTAGACCAGCGGCTTCGCGTAGTCGATGTCGCGAAGCATGACGAAGCAGTCGTCAATCTTAAGGTGCAGATGGGACCTAGCCATCTCGATACCTGCAAGTGCGAGCTCGTGGTACACGGACGCGGGGCAGAGCGGGTGGTCTCCAACGCTATGGCCCATGATGGACTTCCCGAGCTGCGAGATGGGCGTCTCGAAAATTGCGACGCGCTGGTTCGCCGCCGACGGGAACTGGGCCCAGGAGTATAGCATCGAGAAGTTATTGACTAAATCGACGTGCTTCGCGAGCGGGACGGAGGTGGACGCCGATACAACCGCGCCAGGTGCCTCCTCCTTGAACGAGACCCAGAACTTGGACTTCGTGAAGGGGTACGACGGAAGGGAAAGCGTCGAAGGAGAAGAGACATGGCCAAAGACCTCACGCCACCTGAGTTGAATCGGGGACGTGTACAGCTGAGCCAAAGTCGAAGAGACGATTGCCCACGGGTCGTGGTTCTTCTTCAGGGAGCCCAAGAGCATGGTGCTCTTCGAGATCGACGGGTGAACCTTGAACATGGGAAGCGTGATGCTGTGGGGACCAATCTCGATCCATGCGTCGATGTTCGCAAGCTCCGGAATAGCCGCGAGTGAAGTGAGGCCCTTCTCGAAGAGTACTGGCTGCGCGCAGTGGCGAGAGTAGTACTCCGAGTCGAAAACGCCCTCATCGCCGGGCATAACGACCTCACCGGTGACATTGGAGATAATCGGAATAGTGGGCGCGCGGATGGTAACATGCTTAGCAATAGTGGAAAGGTCGTCGAGAAGGGGGTGCATGGCGGAGCTATGGTACCCGAACGGCACCGTCAAGAGGACGTTCTTGCAGCGTACTTCACTGTCGAGGTGTGCCTTCAGTGCCTTGAGCTGAGGAATAGGGCCCGAAACGACGCAGTCAGTGTTGCTGTTAAAGCATGCAACAGAAGTGTCGGGGAAGTCCATCGAAGCTGCCAGCATTTTCGCAACCGCCTCGGAGCCCTGGTTTATCGCGATCATGCCGGTCGTCTCCACGGCGCACTTGCTGACCATGAAGCGGACGCGGTTGGCAATGAGAGTGAGCGCGCCCTTGAGCGTCAAGACGCCAGCGATGACCTGCGCTGCGTACTCGCCCAGACTACAGATAGACGGTCAGAATACTGTCAATTCGAGCGGGAAACCGAACGTACCTGTGGCCTACGACCACCTCGGGAACCAGTCCCCATGACATCCACAACTTGGCGAGCGCGTACTCAAGGGAGAAGATCGCCGCCTGATATGCTTCGAACTCCTCCAGCTGAGTAAGACCACTGGTCTCACCAGCAGGATTGATGATGGCGAGAACGCCCGGGAAGCCGGACGCAGTTAAGATAGCGTGGCACTCGTCGACGGTACGCTTGAAGAGAGGCACGGTCTTGTAGAGGGCAGATCCCATCCCGAGGTACTGGCTGCCCTGGCCAGAGAAGACGAAAGCAACTTTCGCTGGCCGCTCAGTCACAGCGACCTGCGACGCGGCTCTCAACTTCTCGACAAGCTCCTCCTTGGTGCCTGCAGAGACAGCGAGACGCTGGCCGTACAGCTGCCTGCGAGCTGTAGCAGTGTATGCGAAATCGGCGAGGGAGATGGATGCATTCCTGGCATCGCCGAGCCACTCGATGTAACGCATGCGCAGGGCATTGAGTGCCTCGTCCGTCTTCGCTGACAGACCAACGATGAAGGCCGCAGCAGACTCGACCTCAGGTGCCTTCGAGCCCGCCGGGACATATTCCTCCAACAACAGAGCCCCATTGGAACCAGCGGCGCCGAAGTTGTTGAGCATGGCAATTCTCGTCAGTCCCTCTTCCGACGGGTTCCAAGGCGCGTGCTCCCTGTCGATGACCGTGTGATCCTTCTCGAGAGCCACGATCTTCGGGTTCAGATTCTTCAACGAGATCTGGGCGGGAATCGTGCGGTGCTTAAGCATGAGCAGAAGCTTTGCCAGGCCAGCGGAGCCCGACGCAGCCTCGAGATGCCCGATGTTGGCCTTCACGGACGTGATGTGAAGCGGGTTCGCGGGTGTACGGCCGACAGCAAAGACGCCGCGGATACTATCGAGCTCGTTGGGATCGCCAGCCTGCGTGCCAGTGCCGTGAGCTTCGACAACGTTGATGCGACGCGCATCAATGCCAGACTTCTCGAGCGCCTTCTTGAAAAGGATCTGTTGCGTCGGCGAATGCGGATGAGTGATGGAGCTCGCGAGGCCGCTTTGGTTAACCTCAACACCGCGGATGACGCCGAGGATGTTGTCGTCCTCAGCGATGGCATCTGACAGCCGCTTCAGGACGAAAATTCCACAGCCTTCGCTACGCGAGTATCCATCGGCAGAGTCATCGAACGCCTTGCACTGGCCCGTAGGGCTGAGGAAATGACCACGATCTAGACCCATGAACATCTGATACGCCGCACAATTAGTCCAGCCGGTAGAAATTTCAATAAAATGTACGTACGTCGGGAGCAGCGATCACGTTGACACCACCTGCCATGGCCGCAGTGCAGTCCCCGTTCATGAGTGCACGACACGCCTGGTACACGGCGATGATCGAAGACGAGCACGCAGTATCAATGACCACAGATGGACCGCTGAGTTTCATAGCGTATGAAATCCGACCACTGAGGAAGGCGCGCAGAGTGCCTATCCAGAAAATGCGTTAGTTGAGGTAAGAAATTGCCTTCATTTGTCACTCACCAGTGCTGTAATATACGTCAATATCGTTTCGGAGATTCTCAACGTAATCGCCCGTGGCGCAACCGATGTAGCACCCGAACGAGTCCGGCTGGAATGTCGGCGTGGCGTTGGGTACATAACCTGAATCCTCCAGCGCCTCGTACGCGGTGTGAAGCAGCACGCGGCCTTGAGGATCCATGCTGCGTGCCTCACGCGGCGAGATCTTGAAGAACTTGTTGTCAAATGCATCAGGATCGTCGAGGAAGTTGCCCGTATGTGCCTTCATCGACCGGGCACTCTTGGCATCGGCGGGGTTGTTGTAGTCTGAGACCTTGAACCGGTGCTCAGGGATCTGGAGTGATTGACAGGACTATGAGATCGTACGTTCAGGGAGTCTAGGAAAAGAACACTTACCTCCGCAATAGTGTTGATGCCCTTCTCGAGCACCTCCCACAGCTTCTCGACGCTCGGTGCTCCGGGCATGTTAACCGCCATGCCGACAATAGCAATCGGCTCCTGAACGGGAGACATCTTGTTCGCGGCGGAATTCTTCTCCGGGGCAGTCAGATCTACAGACAATGCATTGCGCGTGGGGAATGCCCGCTCCATGCTGCGAGTAAGCCCAGCACCAGGTCCGACGTTGATCAAGCGCACGGCTTCGCCCTCTGGCGCGGCTGACACAAGCGAGCTGACGACGCGATCCCAGTTCACTGGCTGCGTGAGGACCATATCGACGACCGCCTCTACGAACGATCCCTCGCGAGATGAATCGAGCAGCTCGCCAGTGTGCATGGAGCGGACGGGGATCTTGATGTCCGAGAACGAAGGGAACTGAATCCCACGGCGGGACACATCTTCCAGGACCAGATCGCGTGTCGTGCCTGTGTGAATCGGGGAGTGATAAAGGGTATCTACGGTCGTCTTGTGCGGAGGCGCAGAAGTCGGAAGTCGGGACGCGAAGGCAGCGAGAACGTCAGGACGTCCAGATATGGTGACGCAAGTCTCATCCATCACAGCGGTCACGTGCAGAGACTCTGCATTTTCATTCTATGCAGGAATGAATCGAGATAAGCAGGGACATAACAGACGCGCATCAAAGGGTTGTCAGCATCACAATGCGAGGCTAGAACGCGAGGGACTCACCGATTCACGGTACTTGCGCACTGCCTCATCTGCCTCTTGCCGCCCAATCCCCAGGAACACAACGCTCCATGGGAGCGCCGCATCATCTCCGAGACTATTTGCGGATTCAAAGGCTGCGACGCGATAAAGTTGGGCGCGAACTCCGATCCAGAACGCGAGGCGGAATGCCTCGACAGCGTTTGAGATGAACTCCAATGCCGAAACTGAGGTGCCGACGACTGATGCTGGGAGAATACCAGAAGAAAATCCAAGAACACCAAGCTGGTGTGCGAGGTTCTGGGAGAGGATGGCCGCGAAGCGCGGAGACGACGGCGAAGAGTAGGACTCGACGAAGGAGAGGTACCTCAGCGTTTGGATGAGGAAGAGCGTGATCCCAGAGATGACTGGATTGGAGAGGTAGCGCTCCTGAGATAGAGAGAGGAGAGACTCAGGCTTATCGAAGTCCCCAGCTTCGACGCCTGCAGCCTTGCGCTCCTCATCAGTGAATGTAGCGAGCTCTTCTTGGAAACTATCGAAGCAAGAGGAGAGTAGAATAGAGCCTGATGGCGAGGAAGCATCGCGAAGGGCGCGCTCACGGGTCTGGGGTGTGTTGATGGCCGTTGTACCGTGGCCTGCGAAGACCGGGACATTGAAATGCTGCGTATCCGCGATGGAGGACAT | MSSIADTQHFNVPVFAGHGTTAINTPQTRERALRDASSPSGSILLSSCFDSFQEELATFTDEERKAAGVEAGDFDKPESLLSLSQERYLSNPVISGITLFLIQTLRYLSFVESYSSPSSPRFAAILSQNLAHQLGVLGFSSGILPASVVGTSVSALEFISNAVEAFRLAFWIGVRAQLYRVAAFESANSLGDDAALPWSVVFLGIGRQEADEAVRKYRESNENAESLHVTAVMDETCVTISGRPDVLAAFASRLPTSAPPHKTTVDTLYHSPIHTGTTRDLVLEDVSRRGIQFPSFSDIKIPVRSMHTGELLDSSREGSFVEAVVDMVLTQPVNWDRVVSSLVSAAPEGEAVRLINVGPGAGLTRSMERAFPTRNALSVDLTAPEKNSAANKMSPVQEPIAIVGMAVNMPGAPSVEKLWEVLEKGINTIAEIPEHRFKVSDYNNPADAKSARSMKAHTGNFLDDPDAFDNKFFKISPREARSMDPQGRVLLHTAYEALEDSGYVPNATPTFQPDSFGCYIGCATGDYVENLRNDIDVYYSTGTLRAFLSGRISYAMKLSGPSVVIDTACSSSIIAVYQACRALMNGDCTAAMAGGVNVIAAPDMFMGLDRGHFLSPTGQCKAFDDSADGYSRSEGCGIFVLKRLSDAIAEDDNILGVIRGVEVNQSGLASSITHPHSPTQQILFKKALEKSGIDARRINVVEAHGTGTQAGDPNELDSIRGVFAVGRTPANPLHITSVKANIGHLEAASGSAGLAKLLLMLKHRTIPAQISLKNLNPKIVALEKDHTVIDREHAPWNPSEEGLTRIAMLNNFGAAGSNGALLLEEYVPAGSKAPEVESAAAFIVGLSAKTDEALNALRMRYIEWLGDARNASISLADFAYTATARRQLYGQRLAVSAGTKEELVEKLRAASQVAVTERPAKVAFVFSGQGSQYLGMGSALYKTVPLFKRTVDECHAILTASGFPGVLAIINPAGETSGLTQLEEFEAYQAAIFSLEYALAKLWMSWGLVPEVVVGHSLGEYAAQVIAGVLTLKGALTLIANRVRFMVSKCAVETTGMIAINQGSEAVAKMLAASMDFPDTSVACFNSNTDCVVSGPIPQLKALKAHLDSEVRCKNVLLTVPFGYHSSAMHPLLDDLSTIAKHVTIRAPTIPIISNVTGEVVMPGDEGVFDSEYYSRHCAQPVLFEKGLTSLAAIPELANIDAWIEIGPHSITLPMFKVHPSISKSTMLLGSLKKNHDPWAIVSSTLAQLYTSPIQLRWREVFGHVSSPSTLSLPSYPFTKSKFWVSFKEEAPGAVVSASTSVPLAKHVDLVNNFSMLYSWAQFPSAANQRVAIFETPISQLGKSIMGHSVGDHPLCPASVYHELALAGIEMARSHLHLKIDDCFVMLRDIDYAKPLVYNEHVARMVRTSITLDVDGSGSFSVGSQVDGSPEEVHCFGKFKHQSTSKASTKFARVLPIVTRQISSVSSPNDGFAETFSTRTAYEVIFPRVVDYAKEYHTMKTLTVASNGMEAYAIVQLPRDHDRSKFIVHPVFMDTMLHVAGFVANMQGGVNDAYICSKVDTVKAIPALIDNDAEYGVLISNAWVADEGVMLAEAYAVQLKSPGKIVAHLKGMHFRKVRLNSLKRGLAMAAGTSPAHAAPKRAEAPVKKAAPASPMSSKVVSSITFVEPPRDTAPTIDVLAEVTRIVAETCDITASTIQPDGDLEAYGVDSLMSIEIFTKMQSAFPSADLDANVLSSCRNVAQIVAEVSSKFSQEDSGPSTPRTLVTDEKLGEPSVIANFDEVDVKPLLASVLGIGLQEITDDADFESLGLDSLTSIEAHSALQSEYSLTLPTTLFETYTTAKAVNAFLTSQLRPRGKAVEAVKETEVHPSDYKDSDIATAAKVAQIVDGNLNPLVTALRLDSVPVGAQKAKTPGRAPLFLIHDGSGLVNYIQRLSPLDRDIWGIHNPHFITSQPWESVVSMAAEYSEFATKTTSEPLILGWSFGGVVAFEAACQLMKKGVAVKGVLLIDSPTPLAHVPLSDALLESVAKLDGRADTQVGKLVKTQFQMNSRMLGRYDPLAAGGPFPSIVLLRSREGFKPAGVADVPTWLADRSDAQLAISGWERVVGTPIKVIDIPGNHFQPFHTSNIEEVSRRIAEGCAHLESLAA |
| **Pks5** | ATGACTGTATCACCTCCTGCTCCATTCGTACGACCCCTCCTGACGATCGAGCCTGGCTTTGTGGCAACGCGGCGAACTGAACCCGCACAAGGAATCACGACGCTGTACCAGCTGCTGCAGGCTTCCGCAAAGAACAACCCTCAACACATTTACGCCATTCAATTCTCCCACAACAATAAACTTCAAGATGCAGTACAAATCACGCATTCAGACCTGCTTTGCGCAGTCGACACCTGTGCCGAGTGGTTGGTGACTAGGCAGATAGCCCAGCGTTCACGAATGAGATCCGACGACCCACATCAGGTGCAGCGCGGTAGGCCCGTTGCCATATTCCTCGGTAGCGATCTCAACATCATGGTGTACATACTCGCTCTCGTCAAACTCGGAAATCCAGTAAGTGGATCGAATTTTCGCACCTTCGCTGCACGCTCGAAAGAGAGCAGAAAGCTTGCCCGGATGCTGACCTTGCCACGATAATTTTGCGAATGCCGGTTCAGTTTCTCCTCCTGTCAGCAAGGCTTTCCGGAGAGGCCGTCGAGCATCTGCTCAAGGGAGCTGATGCTGGCTTTGTATTGACTGCCGAGCAAGTGTGGGCTAATGCGGGCCTTTCTGCGAGCCATGGTAGCCTTATCAGCGATCTGGACACTCTTACGCATCCTCATTCGAAAGACACGCGCTGCCTTCCGATACCGGCGCCTTCGCTCATATCGTGGCTTGATAGAGAGAAGTGGGCAATGCCAACGAGGCCTAGTCAAGACTCTCTGGAAGGAGAGGAGGTTGATCCAAACGACTGTAGCGTTGTCCTCTTTCATTCAACCGGCTCCACCGGCTTGCCCAAGCTGATTCCACACTGCCACAGATATCTGCTTGGATACGCGAGCTGCCACGAGCTTTCATCCAACCTGGGTGTCGAGCGCGACGTGACCCTGTCGACCCTGCCTCTCTACCATGGCTTTGGTCTGCTTGCCGTCTGCCTATCGCTCTCGATCGGAATGCCGATTGCCCTTCCGGCCGCAACTACGATCCCAAGCGGAGTTTCAGTGGCTCAAATGCTGGCTGAATGCCGTGCTGCGAGGCTTTTCACTGTGCCATCCATCCTGGGTGAAGTACGTGGTCTTGAGTGGCCCGCGGATGATCCTCACTTGTCAATCGCACCTGACAACGAGCCCGGCAAGTCGAGCGGACTGAAGCTGCTTCAAGGCCTCAAGCTCGTCGTTGTGGGAGGTGCGCCCATGCGAACTGAGCTGACAGAATTCCTTGTCGAGCACGGTGTTGAGCTCTTGAACCACTTTGGCGTCACCGAGATCGGAGCGCTCGCCCCCATCGTTCACCCGGGACCTGAGCTTAAATACGACCCCAAGTACCTTCTTATCCGTCGTGACATTCCTCTACGCTTTCGACGAGAGCGGATCCAGAATTCGTCGGACGAGAGCCACCTGCAGATCGGGACTCGGCCGTTTGGATGGACGAAGGATTTCTGGCTCCAGGATTCGCTGGAATACAATCCCAACGCTGGAGGTAATCGGGCGCAAGTACGCATCGAGGGCCGTATCGACGACATTATCGTTCTAGCTACTGGCGAAAAGGTGAACCCTATGGGAATCGAGTCTGCCATCCGACAGCATCCTGCTGTCAGCGATGTGGTCGCCTTTGGCACCAACCGCTTCCAGATCGGTCTTTTGGTGGAGATGCGTTTTGAGCAAGTCACGCCGCCTTCGAGTGATTCTCCGGATCCAGAGTCTCGGAAATGCTCTGCGGAGATCCTAGACATCCTACAAAATGCTAACAGCGCTGCTGACGCCCACGCACAGGTCGATCCGGGCTTGGTACTATTCACTGACCCTGTGGCGAAAGCGTTGCCACGTACGTTCAAGGGCAACGCCCAACGCGCGAGATGTGAAGCAGAATTTGCGCTCGAGATTGAAAAGGCATATGACAGCCTACAGTCAGTCACGGTACAAAGGTCAGCAAATTCACAGGAAGGGACGCACTTCGAAGTCTCCAAGGGAGAAGAGCATCTACGCAAACAGATCGCCGAATTCGTGCAGGAGCAACTGCCTTCGTGTCGCACTATGGGCTACTCGGACTTGCTCGAACGTGACTTCTTCGAGCTCGGTATGGACTCATACAGGGCAGTCAAGCTCGAGCGAAGGCTGAAGCAGTCATTCCCTACGACAGGCGCTTCTTTGATCATGCCAAGCGGCATCGTCTACCGCTTTCCTACCGTAGCAAAGTTGGCAGAAGCGGTGTTATCCTACAAGCGACAGTCAGGACTAGAGACAAATGGTGTCATCCAGCTAGCGCAATGCCAGGAGCAAAGGCTCAATGGCAGCATAGATCAAGCTTGGAGAGCATTTGAAGAAGAGAAGATCCACTTGGAAGAAGTCTCTGCTATGTCTTACGATAGTCCTTGGAAGCTTGATGACAACTCTTGTGGTCGTATCCTCGTTCTCACTGGCTCGACCGGATCACTTGGACGACACTTGCTTAAGACATATCTGCGAGAGCAGCCCAGCCTGGCTCAAGCGTTTTCTAAGATAGTCTGTCTTGTCCGACCTAAGTCAGGCGGAATGGAGAAAGAACATCTTATCGACTGCCTCCGACTTGCAGGAGTAGTTTGGATGCCTGAAGACGAACGCAAGGTCACGATATGGCCATGCGAGCTATCCAGACCAAGACTGGGCCTCACAGCTGCAAGGTACGCTGCTCTCACCCGCAATGCGCTCACGATCCTTCACTGTGCTTGGCCCATGGACTTTCTCCGTCCATTGGAGTCTTTGCAGCCGCACGTCAGCGCTCTGGGCCGACTAGCCTCTCTTGGCCAGTTTGCTGGAGCGAGCTCTCAAGGGCAGCGATCAAGCCTCCTATTTGCTTCATCCATCTCTGTCGTCGCACACTGGAAGGCACGGGATCAAAAGACGCTTGGCACCGTCGTTCCAGAAGAACGGATGGCAGATTTGTCTTCGACAGCTTGTCTGGGATATGCTCATGCAAAGCTTGCTTGCGAGCATCTACTCGAGAAGCTCGATGCACACCTGAAAGCGTCAGGATCCAGCTGCCTAGACAGTGTTGTCGTCCGTATCGGTCAGCTCAGCGGACCCGAGTGTACAGGCGAGTGGACCACGGCCGAACACATGGCGATGATAGCACAGTCGTCCAACACGATTCGAGCGCTTCCAAGGCTCACAGGGGAAGCGTCATGGATACCAGTTGATCGAGCAGCAAAGGCCATGGTCGAGCTAGCCAACGTCAGTCATGGCAGTCGCGAATCCACCAATGCAACATCGACACCAAGTAGAACCGTTCAACATTCTCCACTTGGAGAATGGAGCTCGACGATCGTGGGCTGACATTCTCAAGATTTTTGCTCGGAATATGAACCTCAGCAGCGCCGACACACTCGAGTGGGAAGCATGGCTCGCCTCAGTTCAAAAGCTCGGCAATCAGATCGACGACAGTGTTCCGCAGACGCCAGTAGACGCAAATCCGTGCATCAAGATCCTTGATTTCTTGCAACAAGACTTCTTGCGGCTAGGTACTGGCGGGGTGGTGCTCGATCTGGCCAAAGCCAGGTCCTATTCACGCACTCTCAGGAGCTCCCAAGCAATCAGCGATGAGCTCATCCAGCTCTATCTCGATGGTTGGAAAGGACAGGGCTGGCTTCGATCTCCGATGCGCCGGACTAGCGCCTGA | MTVSPPAPFVRPLLTIEPGFVATRRTEPAQGITTLYQLLQASAKNNPQHIYAIQFSHNNKLQDAVQITHSDLLCAVDTCAEWLVTRQIAQRSRMRSDDPHQVQRGRPVAIFLGSDLNIMVYILALVKLGNPFLLLSARLSGEAVEHLLKGADAGFVLTAEQVWANAGLSASHGSLISDLDTLTHPHSKDTRCLPIPAPSLISWLDREKWAMPTRPSQDSLEGEEVDPNDCSVVLFHSTGSTGLPKLIPHCHRYLLGYASCHELSSNLGVERDVTLSTLPLYHGFGLLAVCLSLSIGMPIALPAATTIPSGVSVAQMLAECRAARLFTVPSILGEVRGLEWPADDPHLSIAPDNEPGKSSGLKLLQGLKLVVVGGAPMRTELTEFLVEHGVELLNHFGVTEIGALAPIVHPGPELKYDPKYLLIRRDIPLRFRRERIQNSSDESHLQIGTRPFGWTKDFWLQDSLEYNPNAGGNRAQVRIEGRIDDIIVLATGEKVNPMGIESAIRQHPAVSDVVAFGTNRFQIGLLVEMRFEQVTPPSSDSPDPESRKCSAEILDILQNANSAADAHAQVDPGLVLFTDPVAKALPRTFKGNAQRARCEAEFALEIEKAYDSLQSVTVQRSANSQEGTHFEVSKGEEHLRKQIAEFVQEQLPSCRTMGYSDLLERDFFELGMDSYRAVKLERRLKQSFPTTGASLIMPSGIVYRFPTVAKLAEAVLSYKRQSGLETNGVIQLAQCQEQRLNGSIDQAWRAFEEEKIHLEEVSAMSYDSPWKLDDNSCGRILVLTGSTGSLGRHLLKTYLREQPSLAQAFSKIVCLVRPKSGGMEKEHLIDCLRLAGVVWMPEDERKVTIWPCELSRPRLGLTAARYAALTRNALTILHCAWPMDFLRPLESLQPHVSALGRLASLGQFAGASSQGQRSSLLFASSISVVAHWKARDQKTLGTVVPEERMADLSSTACLGYAHAKLACEHLLEKLDAHLKASGSSCLDSVVVRIGQLSGPECTGEWTTAEHMAMIAQSSNTIRALPRLTGEASWIPVDRAAKAMVELANIFARNMNLSSADTLEWEAWLASVQKLGNQIDDSVPQTPVDANPCIKILDFLQQDFLRLGTGGVVLDLAKARSYSRTLRSSQAISDELIQLYLDGWKGQGWLRSPMRRTSA |

**Table S4. Primers used in this study.**

| Primer | Sequence 5’-3’ |
| --- | --- |
| HerA-F1 | ccgGAATTCGAGCTCGGTACCATGACCTCCGCTTCTCAGATC |
| HerA-R1 | GACGTCTGAATCGATGTGTGCCTTGAG |
| HerA-F2 | CACACATCGATTCAGACGTCAAATGCAAGAACGTTCTC |
| HerA-R2 | tactacaGATCCCCGGGTACCCTACAACGACTCCAAATATGCACAC |
| Pks5*-*F | tccGAATTCGAGCTCGGTACCATGACTGTATCACCTCCTGCTCC |
| Pks5*-*R | tactacaGATCCCCGGGTACCTCAGGCGCTAGTCCGGCGCATC |

**Table S5. Plasmids constructed in this study.**

| Plasmid | Inserts | Primer 1 | Primer 2 | PCR Template | Vector |
| --- | --- | --- | --- | --- | --- |
| pUARA2-*herA* | 1^st^ half of *herA*  2^nd^ half of *herA* | HerA-F1  HerA-F2 | HerA-R1  HerA-R2 | gDNA of *Hericium erinaceus* | pUARA2 digested with *KpnI* |
| pUSA2-*pks5* | *pks5* | Pks5*-*F | Pks5*-*R | gDNA of *Ustilago maydis* | pUSA2 digested with *KpnI* |
